# Upregulation of microRNA-17-5p contributes to hypoxia-induced proliferation in human pulmonary artery smooth muscle cells through modulation of p21 and PTEN

**DOI:** 10.1186/s12931-018-0902-0

**Published:** 2018-10-10

**Authors:** Guangjie Liu, Peng Hao, Jie Xu, Liming Wang, Yuchuan Wang, Ruifang Han, Ming Ying, Shuangshuang Sui, Jinghua Liu, Xuan Li

**Affiliations:** 1 Department of Respiratory Medicine, Beijing Tongren Hospital, Capital Medical University, Beijing, 100730 China; 2 Tianjin Eye Hospital, Tianjin Eye Institute, Tianjin Key Lab of Ophthalmology and Visual Science, Tianjin, 300020 China; 3 Clinical College of Ophthalmology, Tianjin Medical University, Tianjin, 300020 China; 4 Nankai University Affiliated Eye Hospital, Tianjin, 300020 China

**Keywords:** Hypoxia, Pulmonary hypertension, miR-17-5p, PTEN, p21

## Abstract

**Background:**

Pulmonary arterial smooth muscle cell (PASMC) proliferation in response to hypoxia plays an important role in the vascular remodelling that occurs in hypoxic pulmonary hypertension. MicroRNAs (miRs) are emerging as important regulators in the progression of pulmonary hypertension. In this study, we investigated whether the expression of miR-17-5p is modulated by hypoxia and is involved in the hypoxia-induced proliferation of PASMCs.

**Methods:**

Human PASMCs were cultured under hypoxic conditions. miR-17-5p expression was determined by real-time RT-PCR. A BrdU incorporation assay and time-lapse recording were utilized to determine cell proliferation and migration.

**Results:**

PASMC proliferation was increased by moderate hypoxia (3% oxygen) but was reduced by severe hypoxia (0.1% oxygen) after 48 h. Moderate hypoxia induced miR-17-5p expression. Overexpression of miR-17-5p by transfection with miR-17-5p enhanced cell proliferation and migration in normoxia, whereas knockdown of miR-17-5p with anti-miR-17-5p inhibitors significantly reduced cell proliferation and migration. The expression of miR-17-5p target genes, specifically phosphatase and tensin homologue (PTEN) and cyclin-dependent kinase inhibitor 1 (p21WAF1/Cip1, p21), was reduced under moderate hypoxia in PASMCs. Under normoxia, overexpression of miR-17-5p in PASMCs reduced the expression of PTEN and p21.

**Conclusion:**

Our data indicate that miR-17-5p might play a significant role in hypoxia-induced pulmonary vascular smooth muscle cell proliferation by regulating multiple gene targets, including PTEN and p21, and that miR-17-5p could be a novel therapeutic target for the management of hypoxia-induced PH**.**

## Background

Pulmonary hypertension (PH) is a life-threatening disease characterized by increased pulmonary vascular resistance and pulmonary arterial pressure leading to right heart failure. Chronic obstructive pulmonary disease (COPD) is one of the most common causes of secondary PH. Pulmonary vascular remodelling in COPD is the main cause of the increase in pulmonary artery pressure and is thought to result from the combined effects of hypoxia, inflammation and a loss of capillaries in severe emphysema. The aetiology and pathogenesis of PH are complex, and factors that contribute to vascular smooth muscle cell proliferation play a central role in disease pathogenesis [1]. It is known that hypoxia is a potent stimulus associated with enhanced proliferation of pulmonary artery smooth muscle cells (PASMCs), which is a major contributor to the development of hypoxic PH. Hypoxic PH is characterized by a decreased apoptosis/proliferation ratio in PASMCs and a thickened, disordered adventitia [1, 2].

MicroRNAs (miRNAs) are small noncoding transcripts of 19 to 25 nucleotides that post-transcriptionally regulate gene expression by targeting messenger RNAs (mRNAs). Their expression can be regulated in a transcriptional or post-transcriptional fashion. Following transcription and processing in the nucleus, mature miRNAs downregulate the expression of specific target mRNAs by interacting with the 3′ untranslated region (3’-UTR) of mRNA. MiRNAs regulate gene expression post-transcriptionally by either degrading target mRNAs or inhibiting their translation. It has been estimated that approximately 1,400 distinct miRNAs are encoded by the human genome and that approximately 1/3 of human genes can be regulated by miRNAs [2]. MiRNAs play important roles in the regulation of diverse cellular processes, including proliferation, differentiation, and apoptosis. Aberrant expression of miRNAs is closely associated with pathophysiologic processes, including diabetes, cancer, and cardiovascular disease [3]. Emerging data indicate that hypoxia exerts important modulatory effects on miRNAs at multiple levels of biogenesis [4]. A handful of dysregulated miRNAs have been reported in PH lung specimens from humans and animal models [5].

The miR-17-92 cluster (miR-17, miR-18a, miR-19a, miR-19b, miR-20a, and miR-92a) has been confirmed to be involved in biological development, and miR-17-5p, a member of the miR-17-19 cluster, regulates cell proliferation and migration in various cancers [6–8]. Mir-17-5p has been reported to be upregulated in various cancers and functions as an oncogenic miRNA [9]. It is strongly expressed in embryonic stem cells and has essential roles in vital processes such as cell cycle regulation, proliferation and apoptosis [10, 11]. It has been reported that miR-17-92 is downregulated in PASMCs isolated from patients with PH [12]. In addition, miR-17 and miR-20 have been reported to target bone morphogenetic protein receptor type II (BMPR2) via the STAT3-miR-17/92-BMPR2 pathway [13]. These findings suggest that miR-17 plays an important role in the regulation of PH pathogenesis. However, the mechanism of miR-17-5p modulation in PASMC proliferation in hypoxia is still unclear. In this study, we investigated the role of miR-17-5p in hypoxia-induced cellular responses in human PASMCs. We provide evidence that hypoxia upregulates the transcription of miR-17-5p and that miR-17-5p may modulate PASMC proliferation and migration via targeting phosphatase and tensin homologue (PTEN) and p21. Inhibition of miR-17-5p attenuates hypoxia-induced PASMC proliferation and migration. These data further our understanding of the regulatory function of miR-17-5p on cell proliferation and suggest that miR-17-5p could be developed as a potential therapeutic target for PH.

## Methods

### Cell culture

Human pulmonary arterial smooth muscle cells (PASMCs) were purchased from Lonza. PASMCs were cultured as previously described [14]. Briefly, PASMCs from passages 3–6 were subjected to serum starvation for 24 h before being used for the experiments. The cells were grown to 70% confluence and then incubated in normoxia (21% O_2_) or hypoxia (3% O_2_ or 0.1% O_2_) with 5% CO_2_ for 24–72 h, as described in previously published studies [15, 16].

### BrdU incorporation assay

A BrdU assay was conducted according to the standard protocol of the manufacturer. Cellular proliferation was evaluated by cell count directly before the assay was performed with a kit from Roche that monitors the incorporation of BrdU into newly synthesized DNA. BrdU was detected using an anti-BrdU peroxidase conjugate in accordance with the manufacturer’s instructions. The amount of BrdU incorporated was determined by measuring the absorbance at 450 nm.

### Cell viability evaluation

A cell count was performed using a haemocytometer after trypan blue staining. Cell viability and proliferation were evaluated using thiazolyl blue tetrazolium bromide (MTT, Sigma-Aldrich, Inc., USA) according to the manufacturer’s instructions. MTT reagent was added to each sample and incubated for 3 h to allow the formation of MTT formazan. The resulting formazan was dissolved with dimethyl sulfoxide (DMSO, Sigma-Aldrich, Inc., USA), and the absorbance of each solution was measured at a wavelength of 595 nm with a microplate reader in triplicate (BioTek ELX-800 Absorbance Reader, USA).

### Cell migration assay

Cell migration was monitored with a Cytation 5 Cell Imaging Multi-Mode Reader (BioTek Instruments, Inc., Winooski, VT, USA). Cytation 5 offers image capturing and time-lapse recording. Cells culture inserts (Ibidi GmbH, Martinsried, Germany) were used as barriers to create linear/rectangular gaps (500 μm × 50 μm) in sheets of PASMCs without physical wounding. The insert was then removed using a pair of sterilized forceps with one swift pull. The area devoid of cells was imaged at 2-h intervals until the cells from both sides of the bare area merged. All wound closure assays were performed in quadruplicate.

### Western blot analysis

Cell lysates were prepared with RIPA buffer containing a complete protease mix (Roche). Total protein was assayed for the expression of p21, PCNA, and PTEN by western blot analysis as previously described [14]. Briefly, 50 μg of protein was subjected to SDS-PAGE and electrotransferred to a PVDF membrane. Membranes were processed as described by the manufacturer of the antibodies. The immunoreactive bands were detected by chemiluminescence (Millipore) and quantified by densitometry.

### Real-time reverse transcription PCR

Total RNA was extracted using TRIzol (Invitrogen, Carlsbad, CA, USA). The amount of RNA was quantitatively determined using a Nanodrop spectrophotometer (Thermo Scientific, NJ, USA), and the total RNA was then converted to cDNA with a MultiScribe Reverse Transcriptase Kit (Applied Biosystems, Foster City, CA). The cDNA was quantified using SYBR Green Real-time PCR Master Mix (QPK-201, Toyobo, Tokyo, Japan) in an ABI 7900 PCR system (Applied Biosystems, Foster City, CA). Relative mRNA expression was normalized to GAPDH mRNA expression using the ΔΔCt method.

### Quantification of mature miR-17-5p

Total RNA was extracted from cell samples with a mirVana Isolation Kit (Applied Biosystems, Foster City, CA). Quantitative RT-PCR was performed with a TaqMan microRNA Assay Kit using the manufacturer’s protocol. Relative expression of miRNA was normalized to RNU48 expression using the ΔΔCt method. The primer sequences were as follows: HmiR-17-5p-forward, GCCGCCAAAGTGCTTACA, HmiR-17-5p-reverse, AGAGCAGGGTCCGAGGT; U6-forward, CTCGCTTCGGCAGCACA, U6-reverse, AACGCTTCACGAATTTGCGT.

### PASMC transfection

The miR-17-5p antagomir, the miR-17-5p agomir and their respective negative controls (NC) were obtained from GenePharma (Shanghai, China). Transfection of primary PASMCs was performed using Lipofectamine RNAiMAX Reagent (Invitrogen, Carlsbad, CA) according to the manufacturer’s instructions. The microRNA agomir and antagomir were used in this experiment. The agomir of miR-17-5p used for overexpression was double stranded with the following sequence: sense ACCUGCACUGUAAGCACUUUGTT and antisense CAAAGUGCUUACAGUGAGGUAG. The miR-17-5p antagomir was single stranded with the following sequence: 5’-CUACCUGCACUGUAAGCACUUUG-3′. PASMCs were cultured in six-well plates at 70% confluence at the time of transfection.

### Statistics

The data were tested for normality with Shapiro Wilk’s test or the Kolmogorov-Smirnov test. All quantitative data are reported as the means ± SD. Student’s t-test was used to assess differences between two means. If multiple means had to be assessed, one-way or two-way ANOVA followed by Bonferroni’s post hoc test was performed. If the n number was not sufficient for normality testing, nonparametric tests (the Mann-Whitney U-test or one-way ANOVA followed by Dunn’s test) were used. All statistical analyses were performed using GraphPad Prism (version 7). Statistical significance was defined as *P <* 0.05.

## Results

### Moderate hypoxia increases PASMC proliferation and upregulates miR-17-5p

**Fig. 1:**
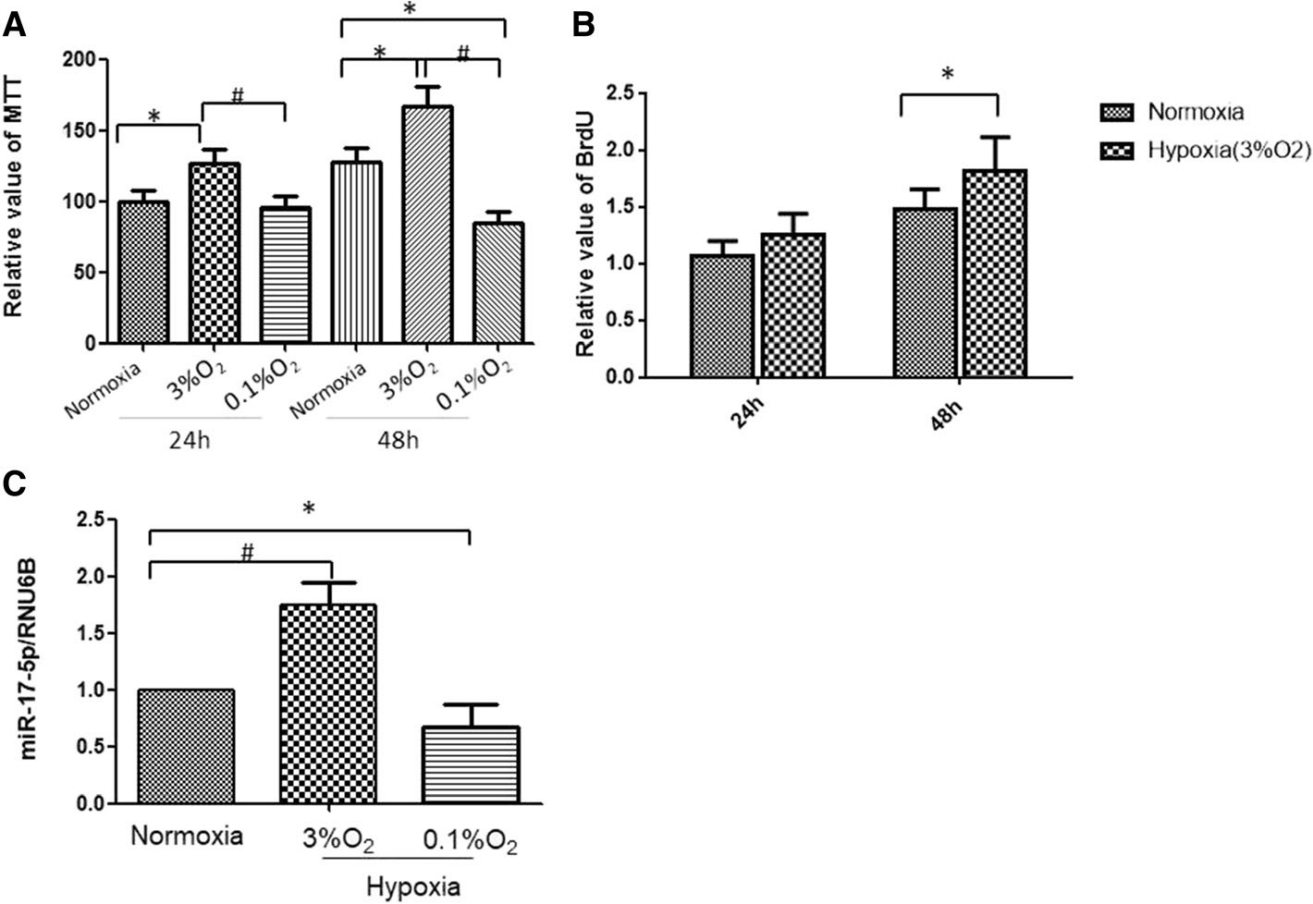
Moderate hypoxia increases PASMC proliferation and upregulates miR-17-5p. **a** Cell viability was assayed by MTT assay after PASMCs were incubated under hypoxic conditions for 24 h and 48 h. **b** Cell proliferation was examined by BrdU assay. Moderate hypoxia (3% O2) stimulated PASMC proliferation. The results are the means ± SD of three independent experiments, and in each experiment, three parallel samples were analysed per group. **c** miR-17-5p levels were quantified by real-time PCR and normalized to RNU48 expression after hypoxia stimulation for 48 h. The results are the means ± SD, and the experiments were performed in triplicate and were repeated a minimum of three times. *P < 0.05, #*P* < 0.01

Hypoxia plays an important role in regulating vascular smooth muscle cell proliferation. We examined the effects of oxygen tension on the proliferation of PASMCs, and our data showed that moderate hypoxia (3% oxygen) significantly increased cell proliferation as assessed by both the MTT assay and the BrdU assay (Fig. 1a and b). Figure 1b shows that moderate hypoxia induced a 26% increase in cell proliferation after 24 h and a 30% increase after 48 h compared with normoxia. However, severe hypoxia (0.1% O_2_) significantly decreased cell viability at 48 h (Fig. 1a). Therefore, we investigated the effect of moderate hypoxia (3% O_2_) in the following study.

To explore the role of miR-17-5p on PASMC proliferation in response to hypoxia, we performed qRT-PCR to detect the levels of miR-17-5p in PASMCs under hypoxic conditions. Compared with the expression under normoxia, the expression of miR-17-5p was significantly increased under moderate hypoxia in PASMCs (Fig. 1c). In addition, severe hypoxia (0.1% oxygen) significantly reduced miR-17-5p expression in PASMCs.

### miR-17-5p plays a role in PASMC proliferation and migration

**Fig. 2:**
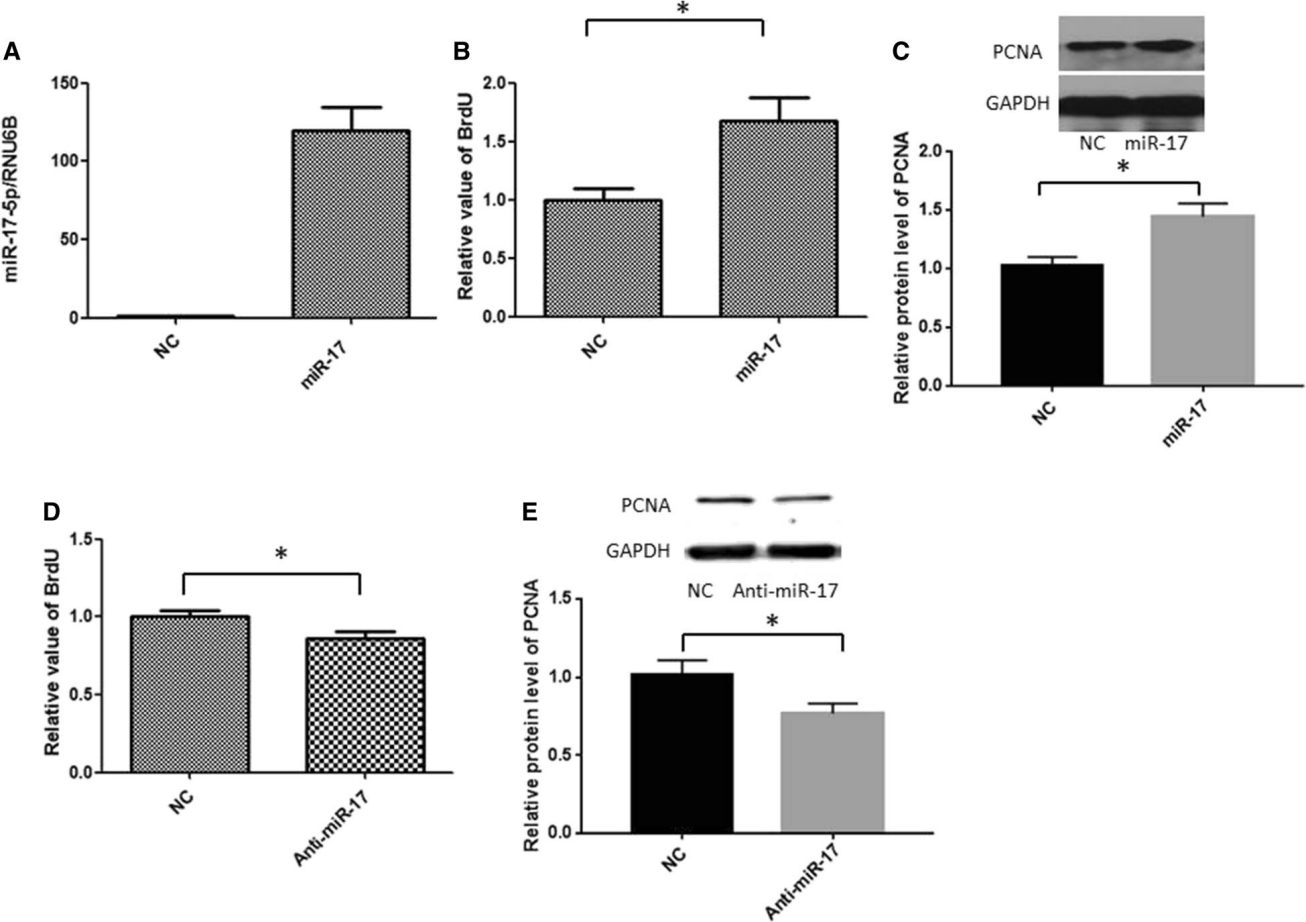
miR-17-5p plays a role in PASMC proliferation. PASMCs were transfected with an miR-17-5p agomir (miR-17-5p), an miR-17-5p inhibitor (anti-miR-17-5p), or a negative control (NC). **a** miR-17-5p expression. The results are the means ± SD, and the experiments were performed in triplicate and were repeated a minimum of three times. **b** Overexpression of miR-17-5p increased cell proliferation. The results are the means ± SD of three independent experiments, and in each experiment, three parallel samples were analysed per group. **c** Protein level of PCNA. The representative blots are shown in the upper panel, and the quantification is shown in the lower panel. The results are the means ± SD of three separate experiments. **d** Inhibition of miR-17-5p reduced PASMC proliferation. The data are shown as the means ± SD of three independent experiments, and in each experiment, three parallel samples were analysed per group. **e** The expression of PCNA was analysed by western blot analysis and normalized to GAPDH expression. The data are expressed as the means ± SD (*n* = 4). *P < 0.05, #*P* < 0.01

Since vascular SMC proliferation and migration are known to contribute to vascular remodelling in PH, we investigated whether miR-17-5p plays a relevant role in the regulation of PASMC proliferation and migration. We transfected PASMCs with miR-17-5p or its negative control (NC) and studied the effects of overexpressing miR-17-5p on PASMC proliferation and migration. As shown in Fig. 2a, transfection of miR-17-5p substantially increased miR-17-5p expression. Overexpression of miR-17-5p resulted in increased cell proliferation compared with that of controls, as measured by the BrdU incorporation assay (Fig. 2b). Moreover, overexpression of miR-17-5p induced proliferating cell nuclear antigen (PCNA) expression (Fig. 2c). Densitometric measurements of western blots showed that miR-17-5p overexpression significantly increased PCNA protein levels by ~ 50%. To further confirm the effect of miR-17-5p on PASMC proliferation, we transfected cells with an miR-17-5p antagomir to inhibit miR-17-5p. Treatment with the miR-17-5p inhibitor reduced PASMC proliferation (Fig. 2d). Additionally, the expression of PCNA was decreased after transfection of PASMCs with the miR-17-5p antagomir (Fig. 2e).

**Fig. 3:**
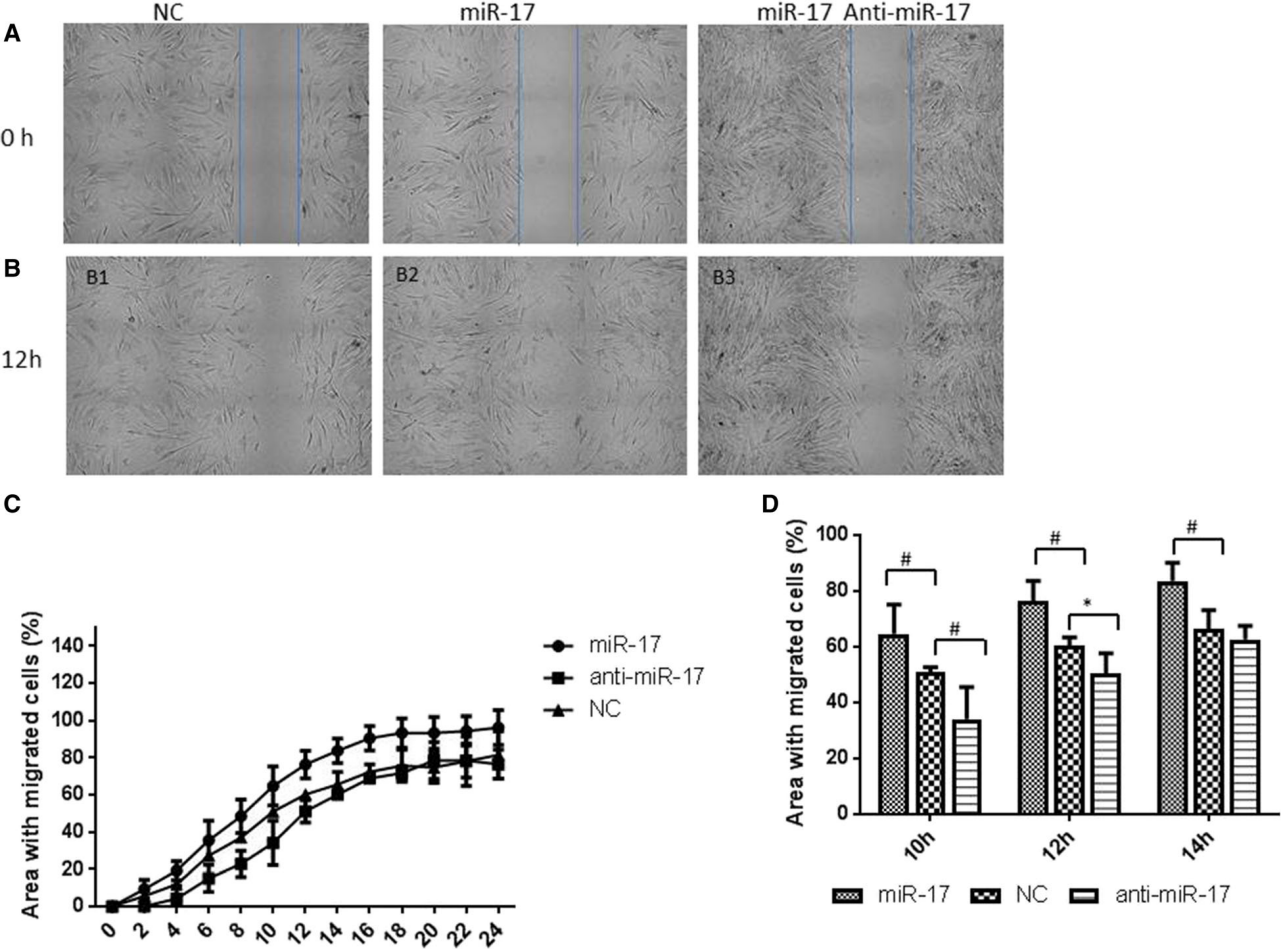
miR-17-5p plays a role in PASMC migration. **a**-**d** miR-17-5p overexpression led to increased PASMC migration as measured by time-lapse microscopy. PASMCs were cultured in Ibidi culture inserts. Compared with the negative controls, cells overexpressing miR-17-5p showed a 16% increase in migration at 12 h (B1-B2). Compared with the control treatment, inhibition of miR-17-5p via transfection with an miR-17-5p antagomir caused a 10% decrease in migration at 12 h (B1, B3). **c** Change in the cell-covered area over time. **d** PASMC migration after 10 h, 12 h, and 14 h. The values are expressed as the means ± SD of three independent experiments, and in each experiment, three parallel samples were analysed per group. *P < 0.05, #*P* < 0.01

To investigate the role of miR-17-5p in PASMC migration, we used a non-traumatic migration assay under a time-lapse microscope. As shown in Fig. 3, the upregulation of miR-17-5p promoted PASMC migration compared to the migration of control cells. MiR-17-5p overexpression induced an ~ 16% increase in the cell-covered area after 12 h, whereas the downregulation of miR-17-5p expression inhibited migration by ~ 10% after 12 h (Fig. 3a, b, c and d). These results indicated that miR-17-5p plays a role in PASMC proliferation and migration.

### miR-17-5p regulates the expression of proteins involved in cell cycle progression, cell proliferation and apoptosis in PASMCs in vitro

**Fig. 4:**
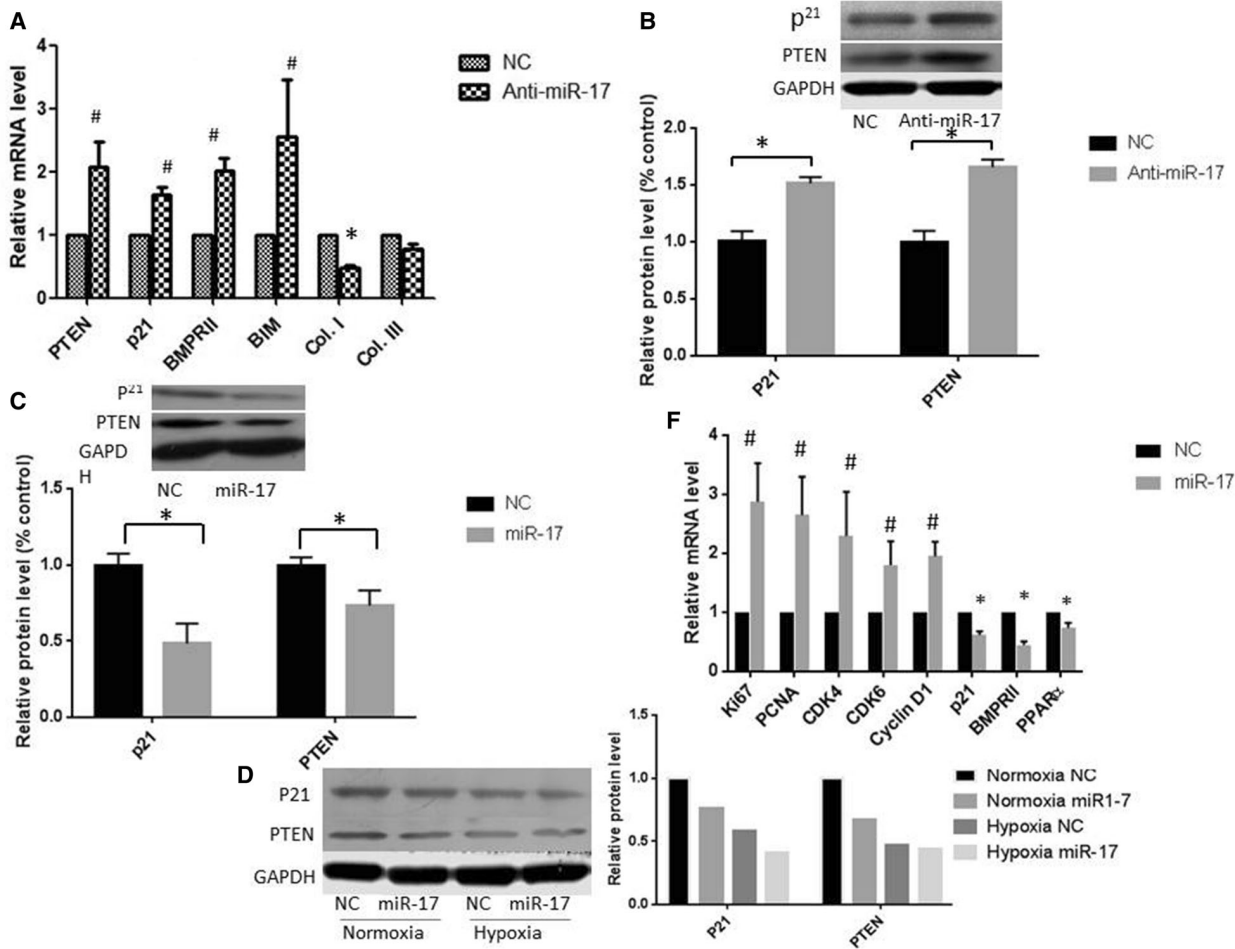
miR-17-5p regulates the expression of proteins involved in cell cycle progression, cell proliferation and apoptosis in PASMCs in vitro. PASMCs were transfected with miR-17-5p agomir (miR-17-5p) or miR-17-5p antagomir (anti-miR-17-5p) oligonucleotides. mRNA levels of four target genes of miR-17-5p (**a**). The expression levels of two target genes of miR-17-5p were determined by western blot (**b**‑**d**). mRNA levels of five genes involved in cell cycle progression, cell proliferation and apoptosis in PASMCs (**f**). The results are expressed as the means ±SD of at least three independent experiments, and qRT-PCR was conducted in triplicate. *P < 0.05, #*P* < 0.01

PTEN, p21, BIM (Bcl-2-like protein 11, Bcl2l11), and BMPR2 have been reported as miR-17-92 cluster targets [13, 17–19]. We found that the inhibition of miR-17-5p by transfection with an miR-17-5p inhibitor caused the upregulation of PTEN, p21, BIM, and BMPR2 in PASMCs at the mRNA level. Western blot analysis also showed that the protein levels of PTEN and p21 were increased by transfection with the miR-17-5p inhibitor (Fig. 4a and b). In contrast, overexpression of miR-17-5p in PASMCs resulted in the downregulation of PTEN and p21 at the protein level under normoxic or hypoxic conditions (Fig. 4c and d). Importantly, further studies showed that transfection of an miR-17-5p agomir into PASMCs induced the expression of proliferation-related genes, including Cyclin D1, Cyclin-dependent kinase (CDK) 4, CDK6, Ki67, and PCNA (Fig. 4f). These results indicated that miR-17-5p could mediate PASMC proliferation by regulating its targets, PTEN, BMPR2, and p21, which negatively control cell growth. In addition, inhibition of miR-17-5p reduced the expression of collagen type I and collagen type III in PASMCs, suggesting the potential of miR-17-5p to act as a target for the prevention of pulmonary vascular remodelling.

### Effect of hypoxia on miR-17-5p predicted targets

**Fig. 5:**
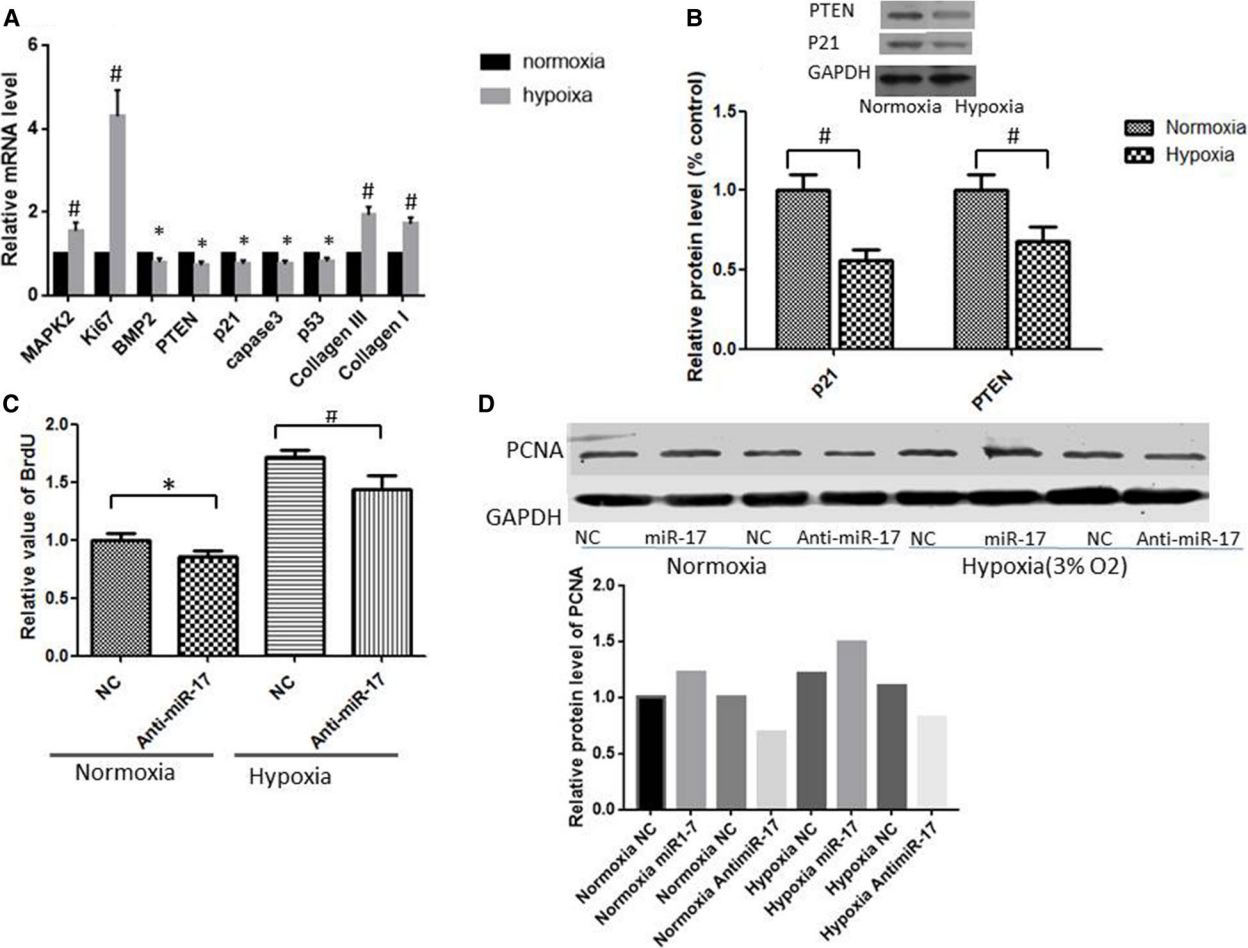
Hypoxia reduces the expression of miR-17-5p predicted targets. PASMCs were exposed to normoxic or hypoxic conditions for 48 h. **a** mRNA levels of the genes involved in proliferation and apoptosis in PASMCs under hypoxic or normoxic conditions. **b** Hypoxia reduces the protein expression of validated miR-17-5p targets, including PTEN and p21. **c** When miR-17-5p was inhibited, PASMC proliferation was significantly decreased under both normoxia and hypoxia. The results are expressed as the means ± SD of at least three independent experiments, and qRT-PCR was conducted in triplicate. **d** PCNA expression was examined by western blot analysis and normalized to GAPDH expression. *P < 0.05, #P < 0.01

Moderate hypoxia increases PASMC proliferation by regulating some genes involved in cell proliferation and apoptosis. Gene expression profiling was used to identify hypoxia-modulated genes. As shown in Fig. 5a, we found that moderate hypoxia induced the upregulation of pro-proliferative genes, including ki67 and MAPK2; several anti-proliferative genes, including p21, BMP2, and PTEN, were downregulated. Exposure to moderate hypoxia caused PASMC proliferation accompanied by the upregulation of miR-17-5p and the downregulation of p21, PTEN, and BMP2. The results showed that the expression of miR-17-5p was negatively correlated with the levels of anti-proliferation genes, including p21, PTEN, and BMP2, in PASMCs under hypoxia stimulation. Among these genes, both PTEN and p21 have been identified as miR-17-5p target genes. We therefore measured the protein levels of these two miR-17 targets in cell lysates obtained from PASMCs exposed to normoxia or hypoxia. As shown in Fig. 5b, hypoxia significantly reduced the protein levels of p21 and PTEN. In addition, inhibition of miR-17-5p reduced cell proliferation in both normoxia and hypoxia (Fig. 5c). In accordance with the results of the BrdU assay, PCNA protein expression, as determined by western blot analysis, was increased in PASMCs under hypoxia stimulation (3% O2). Furthermore, the expression of PCNA was increased in PASMCs when miR-17-5p was overexpressed using the miR-17-5p agomir. On the other hand, inhibition of miR-17-5p by the miR-17-5p antagomir transfection decreased PCNA expression in PASMCs (Fig. 5d). These results suggested that miR-17-5p might play an important role in mediating hypoxia-induced PASMC proliferation and migration via targeting p21 and PTEN.

## Discussion

The miR-17-92 cluster is located in the locus of the non-protein-coding gene MIR17HG (the miR-17/92 cluster host gene, also known as C13orf25) [20]. The miR-17-92 cluster has been well studied in various cancer cells. It is known that C-MYC is involved in activating MIR17HG transcription. The miR-17-92 cluster is important in cell cycle progression, proliferation, apoptosis and other pivotal processes [9, 21]. The miR-17-92 cluster has been linked to vascular pathogenesis, including that of coronary artery disease and PH [5, 12]. However, the function of this locus in PASMCs is still unclear. In the present study, we demonstrated that miR-17-5p, a single miRNA from this locus, induces PASMC proliferation and migration. Previous studies have indicated that miR-17-5p is often upregulated in various cancers and is correlated with cell cycle progression, apoptosis and proliferation [11, 22]. Here, we found that miR-17-5p was upregulated in PASMCs by hypoxia stimulation. Hypoxia is a well-known stimulus for the development of PH. Hypoxia promotes pulmonary arterial wall remodelling via the induction of cell proliferation in all three layers, particularly in smooth muscle cells. Our data provide evidence that hypoxia induces the upregulation of miR-17-5p in PASMCs. miR-17-5p promotes PASMC proliferation and migration, while inhibiting miR-17-5p attenuates PASMC proliferation and collagen synthesis. These findings are consistent with those of a previous study reporting that the miR-17-92 cluster can induce both PASMC proliferation and differentiation and that SMC-specific knockout of miR-17-92 attenuates hypoxia-induced PH in mice [12]. However, the roles of miR-17-5p in PASMCs may be more complex than previously assumed, and further in vivo functional studies are needed to establish its specific role in PH pathogenesis.

The expression patterns of miRNAs are regulated by many factors, among which hypoxia is well known to alter the expression of a number of miRNAs [4, 23]. Caruso et al. reported that chronic hypoxia reduced the expression of Dicer. Dicer is involved in miRNA processing and may cause the downregulation of a number of miRNAs in rat PH induced by hypoxia and monocrotaline (MCT) [24]. Abnormal miR-17-92 expression in PH has been reported in previous studies [12, 13, 25]. Chen T and coworkers reported that miR-17-92 is downregulated in PASMCs isolated from patients with PH, and the reduced expression of miR-17-97 is associated with decreased levels of the dedifferentiated smooth muscle cell phenotype [12]. Pullamsetti and colleagues reported that miR-17 is transiently upregulated in the hypoxia-induced PH mouse model [25]. Consistent with this report, miR-17-5p was found to be upregulated under hypoxia at 48 h in PASMCs in vitro in this study. These results implied that the regulation of miRNAs by hypoxia might be different according to the details of the experimental conditions, cell types, and both the duration and the degree of reduced oxygen tension. Our results demonstrate that miR-17-5p expression increases after acute hypoxia in PASMCs in vitro and that overexpression of miR-17-5p promotes PASMC proliferation. Therefore, it appears that the miR-17 cluster is upregulated in the initial stage and serves as a promoter for vascular smooth muscle cell proliferation during the remodelling phase. Further studies should investigate the time course of miR-17-5p expression in PH models in vivo.

Hypoxia is thought to be a cause of pulmonary vascular remodelling and PASMC proliferation. Cell cycle progression is dependent on the expression and activation of specific CDK enzymes, which form complexes with their regulatory subunits, the cyclins. The cyclin-CDK complexes formed in cell cycle progression are regulated by CDK inhibitors, such as p21/Cip1 and p27/Kip1. p21 is a cell cycle inhibitor that inhibits the activity of cyclin-CDK2, CDK1, and CDK4/6. Current research implies that p21 may represent a major target for new therapies targeting PASMC proliferation during the progression of PH [26]. Moderate hypoxia has been found to enhance the proliferation of PASMCs via the p21 pathway, whereas severe hypoxia (0.1% oxygen) leads to cell cycle arrest via the p53-p21 pathway [27]. Consistent with previous reports, our present study demonstrated that 3% oxygen induced PASMC proliferation and migration accompanied by decreased expression of p21. In addition, we found that 3% oxygen induced the expression of miR-17-5p. The miR-17-92 cluster has been reported to be a target for p53-mediated transcriptional repression under severe hypoxia [16]. In line with this finding, we found that 0.1% oxygen reduced miR-17-5p expression. These results suggest that miR-17-5p is involved in mediating hypoxia-induced cell proliferation and apoptosis. The miR-17-5p target p21 may regulate moderate hypoxia-induced cell proliferation.

We demonstrated that moderate hypoxia caused PASMC proliferation accompanied by the upregulation of miR-17 and downregulation of p21, PTEN, and BMP2. It has been reported that PTEN, p21, and BMPRII are the targets of the miR-17-92 cluster [17, 18, 28]. There is evidence indicating that miR-17 represses BMP signalling by targeting BMPRII [28]. PTEN and p21 have been identified as anti-proliferation genes. Furthermore, it has been reported that BMP2 inhibits cell proliferation via p21 in human aortic smooth muscle cells. Additionally, BMP2 has been reported to induce PTEN expression in PASMCs under hypoxia [29–31]. Our results are consistent with the findings of these studies.

The migration of proliferating vascular smooth muscle cells is a crucial factor in PH. In this study, we found that miR-17-5p plays an important role in the migration of PASMCs. Similar studies in gastric cancer cells [32] have shown that miR-17-5p regulates the gastric cancer cell line SGC-7901 in vitro. These results suggest that the upregulation of miR-17-5p in hypoxia is important for both PASMC proliferation and migration. Based on reports in the published literature, we examined the effect of hypoxia on p21 and PTEN, which have been reported to be miR-17-5p targets related to cell proliferation and migration [17, 18, 33]. We found that hypoxia reduced the expression of both p21 and PTEN. Under normoxia, overexpression of miR-17-5p caused decreased expression of these proteins, and knockdown of miR-17-5p increased their expression, suggesting that they are targeted by miR-17-5p in PASMCs.

PTEN, a tumour suppressor, has been shown to play a crucial role in the regulation of adhesion, migration, growth and apoptosis. Jianhua Huang and Christopher D. Kontos reported that overexpression of PTEN significantly inhibited PASMC proliferation and migration [34]. Consistent with these results, we found that decreased expression of PTEN was correlated with increased proliferation and migration under hypoxia or after transfection with miR-17-5p.

We examined the mRNA levels of cell proliferation-related genes in PASMCs transfected with an miR-17-5p antagomir or agomir. Consistent with the constitutive expression of miR-17-5p being potentially oncogenic [9, 35], the mRNA expression of pro-proliferative genes (ki67, PCNA, cyclin D1, CDK4, and CDK6) was increased in miR-17-5p-overexpressing PASMCs, representing a proliferative signal. Inhibition of miR-17-5p significantly upregulated the mRNA levels of genes including p21, PTEN, and BMPR2, which are confirmed as targets of miR-17-5p. The suppression of these target genes is consistent with the pro-proliferative phenotype observed in PASMCs overexpressing miR-17-5p.

## Conclusion

In summary, miR-17-5p, a single miRNA, could drive a proliferative phenotype in PASMCs. Moderate hypoxia increases the expression of miR-17-5p, which is involved in cell proliferation and migration via the targeting the PTEN and p21 genes in PASMCs. Inhibition of miR-17-5p attenuates hypoxia-induced PASMC proliferation and migration. These data further our understanding of the regulatory function of miR-17-5p on cell proliferation and suggest that miR-17-5p could be developed as a potential therapeutic target for PH.
